# Chlorophyll and Chlorophyll Derivatives Interfere with Multi-Drug Resistant Cancer Cells and Bacteria

**DOI:** 10.3390/molecules24162968

**Published:** 2019-08-16

**Authors:** Erjia Wang, Markus Santhosh Braun, Michael Wink

**Affiliations:** Institute of Pharmacy and Molecular Biotechnology, Heidelberg University, INF 364, 69120 Heidelberg, Germany

**Keywords:** multi-drug resistance, synergism, efflux pumps, antibiotic susceptibility, photosensitization

## Abstract

Multidrug resistance (MDR) causes challenging tasks in medicine. Human cancer cells, as well as microorganisms, can acquire multiresistance due to the up-regulation of efflux pumps (ABC transporters) and are difficult to treat. Here, we evaluated the effects of chlorophyll, the most abundant pigment on the globe, and its derivative, pheophytin, on cancer cells and methicillin-resistant *Staphylococcus aureus* (MRSA). We found that both substances have significant reversal effects on multidrug-resistant CEM/ADR5000 cells (RR_pheophytin_ = 3.13, combination index (CI)_pheophytin_ = 0.438; RR_chlorophyll_ = 2.72, CI_chlorophyll_ < 0.407), but not on drug-sensitive CCRF-CEM cells when used in combination with doxorubicin. This indicates that the porphyrins could interact with efflux pumps. Strong synergism was also observed in antimicrobial tests against MRSA when combining ethidium bromide with chlorophyll (FICI = 0.08). As there is a strong need for new drugs in order to reliably treat MDR cells, our research provides potential candidates for further investigation.

## 1. Introduction

Multi-drug resistance (MDR) is one of the major challenges in cancer therapy and is mainly mediated by the over-expression of ATP-binding cassette (ABC) transporter proteins. The drug efflux protein P-glycoprotein (P-gp) is a major member of the ABC transporter superfamily. P-gp acts as an energy-dependent drug efflux pump and reduces the intracellular concentration of structurally unrelated drugs inside the cells. These drugs are mostly lipophilic and have entered the cell by free diffusion [1,2]. Hence, inhibiting P-gp could be one approach to re-sensitize resistant tumor cells and overcome MDR.

Apart from cancer therapy, MDR also plays a cardinal role in the treatment of infectious diseases. Just like tumor cells, some bacteria express efflux systems that have the capacity to extrude cell-borne toxins and noxious environmental agents (including antibiotics) and thereby prevent cell damage. Consequently, an up-regulated active drug export results in antibiotic resistance [3]. The efflux pumps of bacteria, which are responsible for multi-drug resistance, are also members of the ABC transporter superfamily. Accordingly, it has been shown that some gram-positive bacteria contain efflux pumps that are affected by agents that inhibit P-gp of cancer cells [4,5]. For example, the pump NorA of *Staphylococcus aureus* extrudes ethidium bromide, and the resistance is reversed by verapamil, a calcium channel antagonist, which can inhibit MDR pumps of mammalian cancer cells [5,6]. Ethidium bromide is a well-known substrate of most efflux pumps and is widely used to detect the efficiency of efflux pumps by means of assessing fluorescence under ultraviolet light [7,8,9].

However, microbes do not only pose a risk to mammals and other animals, but also to plants. Plants are targeted by the same biotic and abiotic stress factors but, in contrast to the majority of animals, are restricted to the same location and cannot evade threats by flight. Nor do plant have an adaptive immune system. Accordingly, there is a strong selective pressure leading to the evolution of a diverse repertoire of plant defense strategies, such as the expression of polyphenols, antimicrobial peptides, and other secondary metabolites. Those are mediated by the selective up-regulation of resistance genes and are effective in reducing damages subsequent to bacterial, fungal, or viral infection [10]. Many plant drugs have already been shown to possess antiproliferative and antimicrobial activities, which are mediated by an inhibition of ABC transporters in MDR bacteria [11,12].

However, evidence on whether or not chlorophyll, the most abundant pigment around the globe, plays a role in pathogen defense of plants is still rare. Chlorophyll plays a key role in the energy metabolism of green plants (photosynthesis) by absorbing photo energy and converting it into chemical energy. Nevertheless, its light-absorbing properties also render chlorophyll a potential phototoxin upon irradiation [13].

Spinach, a chlorophyll-rich vegetable and part of our daily diet, is well known for its multiple biofunctions such as its antioxidant, antiaging, anticancer, and antimutagenic activity [14,15,16,17,18,19,20]. Some scientists suggested that chlorophyll-related compounds may play a critical role in the wholesomeness of green vegetables. For instance, chlorophyll was reported to reduce the formation of cytotoxic haem metabolites and decrease colon cancer risk [21]. Also, pheophorbide-related compounds originating from the breakdown of chlorophyll demonstrated anticancer activities against a panel of human tumor cell lines, including lung carcinoma (A549), ileocecal carcinoma (HCT-8), kidney carcinoma (CAKI-1), and breast adenocarcinoma (MCF-7), which are mediated by direct photoirradiation [22]. In addition, pheophorbide and pyropheophorbide are known efflux pump inhibitors which affect antibiotic resistance in bacteria [23]. However, whether or not chlorophyll has the ability to reverse multi-drug resistance in cancer cells and bacteria has, to date, not been thoroughly investigated.

In this study, multi-drug resistant P-glycoprotein-overexpressing CEM/ADR 5000 leukemia cells and its parent cell line, T-cell lymphoma CCRF-CEM, were used to investigate the effect of chlorophyll and pheophytin alone and in combination with doxorubicin on the reversal of P-gp mediated MDR. The pathogenic microorganisms *Staphylococcus aureus* ATCC 25,923 and MRSA NTCT 10,442 were selected to study the effects of chlorophyll and pheophytin on microorganisms.

## 2. Results

### 2.1. Cytotoxicity of Chlorophyll and Pheophytin in Human Cancer Cells

The cytotoxicity of chlorophyll and pheophytin was determined against the Human T-cell lymphoma CCRF-CEM and its resistant subline, CEM/ADR5000 by MTT assay. As shown in Table 1, the IC_50_ value of chlorophyll indicates its limited cytotoxicity against CCRF-CEM. It can even be regarded as non-cytotoxic against CEM/ADR5000 cells, whereas pheophytin showed moderate cytotoxicity against these two cell lines. Since a prerequisite for an appropriate chemosensitizer is low toxicity to human cells, chlorophyll and pheophytin might be promising candidates for further study.

### 2.2. MDR Reversal Assay in CEM/ADR 5000

**Figure 1:**
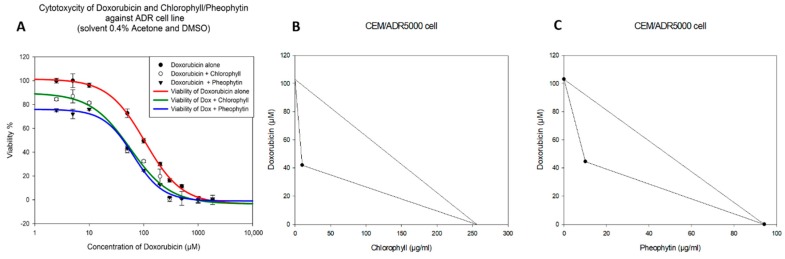
(**A**) is the synergistic cytotoxicity of doxorubicin in combination with chlorophyll (10 µg/mL) and pheophytin (10 µg/mL) in CEM/ADR 5000 doxorubicin-resistant cell line. (**B**) (chlorophyll) and (**C**) (pheophytin) are isobologram analyses of interactions between doxorubicin and chlorophyll or pheophytin. All dots are clearly located below the line of additivity, suggesting synergism.

A non-toxic concentration was chosen for the combination with doxorubicin. Table 2 shows the IC_50_ values of doxorubicin alone and two-drug combinations in CEM/ADR 5000 cells and CCRF-CEM cells. In addition, the reversal ratio and the combination index (CI) are shown. CI analysis provides qualitative information on the nature of drug interaction and a quantitative measure of the extent of drug interaction [24]. Briefly, CI values < 1 represent synergism, 1 means additive effects, and >1 is regarded as antagonism. As shown in Table 2, both chlorophyll and pheophytin can significantly decrease the IC_50_ values of doxorubicin against CEM/ADR 5000 cells. Both the CI values and isobologram analysis (Figure 1A–C) demonstrated synergistic effects of doxorubicin with chlorophyll and pheophytin. Since, due to its non-cytotoxicity, the IC_50_ value of chlorophyll in resistant CEM/ADR 5000 cell line was difficult to determine, the highest concentration tested was used to construct the line of additivity. As can be seen from concave isoboles, both chlorophyll and pheophytin exhibited synergy with doxorubicin. Additionally, the reversal ratio confirmed that chlorophyll and pheophytin could significantly enhance the cytotoxicity of doxorubicin in a synergistic manner. As expected, the parental sensitive line CCRF-CEM remained completely unaffected in synergism experiments (Table 2).

### 2.3. Selection of Bacteria for Antimicrobial Susceptibility Testing

**Figure 2:**
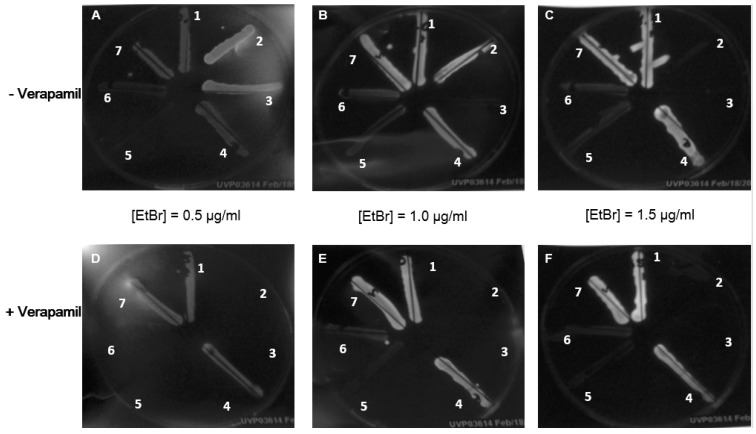
Evaluation of efflux pump activity based on bacterial cultures growing on ethidium bromide agar plates (cartwheel method). Cultures were swabbed on Müller Hinton blood plates containing increasing concentrations of ethidium bromide (0.5–1.5 µg/mL) without (**A**–**C**) and with (**D**–**F**) the addition of 200 µg/mL verapamil. Fluorescence was detected under UV light after 16 h of incubation at 35 °C. 1: *E. coli* ATCC 25922, 2: MRSA NCTC 10442, 3: *S. aureus* ATCC 25923, 4: *B. cepacia* ATCC 25414, 5: *E. faecalis* VRE ATCC 51299, *E. faecalis* ATCC 29212, and 7: *E. coli* ATCC 35150.

In order to find an appropriate bacterial strain for the further investigation of effects on the efflux pumps, the ethidium bromide agar cartwheel method was applied to screen 7 different strains. Three gram-negative bacteria, *E. coli* ATCC 25,922 (Figure 2, strain number 1), *E. coli* ATCC 35,150 (Figure 2, strain number 7), *B. cepacia* ATCC 25,414 (Figure 2, strain number 4), as well as the two strains of *E. faecalis* (Figure 2, strain numbers 5 and 6) were able to grow under all conditions, including the highest concentration of ethidium bromide and ethidium bromide plus verapamil. The comparison of the different levels of ethidium bromide incorporated into the agar medium shows a positive correlation between ethidium bromide concentration and fluorescence intensity. From Figure 2, it is evident that bacteria exposed to verapamil display a relatively stronger fluorescent signal when compared to bacteria growing on verapamil-free media. Since the strains of *Staphylococcus aureus* exhibited the most pronounced response to verapamil exposition and were sensitive to low ethidium bromide concentrations, MRSA NTCT 10,442 (Figure 2, strain number 2), was selected for the further experiments combining ethidium bromide and chlorophyll.

### 2.4. Determination of Minimum Inhibitory Concentrations of the Single Substances

As seen in Table 3, under dark conditions, chlorophyll and its derivative showed no antimicrobial activity against gram-positive and gram-negative bacteria, while the calcium channel blocker verapamil exhibited moderate antibacterial potential in vitro. On the other hand, the porphyrins exhibited a significantly augmented inhibitory effect when excited by visible light. The emission wavelengths of the light source used were between 400 to 750 nm. The emission peaks ranged from 420–480 and 500–700 nm and were similar to the absorption peaks of chlorophylls.

### 2.5. Synergism Test Using MRSA

Whether or not light affects porphyrins’ potential to act as efflux pump inhibitors was tested for in checkerboard assays using chlorophyll and ethidium bromide. As seen in Table 4, under light conditions, chlorophyll not only inhibited the growth of MRSA when applied alone, it also showed a marked synergism with ethidium bromide. On the contrary, without illumination, no effect was evident.

## 3. Discussion

In the present investigation, we demonstrated adjuvant anticancer properties of chlorophyll and pheophytin isolated from spinach. These effects are most likely due to the MDR reversal activities of chlorophyll and pheophytin in multi-drug resistant P-glycoprotein-overexpressing cells such as CEM/ADR 5000. This is in line with previous findings on several antiproliferative functions, such as the anticancer activities of pheophorbides against several human cancer cell lines [22]. Also, pheophorbide *a* has proven to be a photosensitizer, which after irradiation causes a photodynamic effect on in human cancer cells based on the induction of lipid peroxidation [25]. Moreover, some research concluded that the porphyrins might be the functional structure of chlorophyll derivatives, which result in anticancer effects [14]. To our knowledge, this is the first study to show that chlorophyll and pheophytin, in combination with other chemotherapeutic agents, overcome multi-drug resistance in cancer cells. Given the synergistic effects of chlorophyll and pheophytin with doxorubicin on P-gp overexpressing cells, the fact that they do not influence non-resistant cells (Table 2), and their absent/low intrinsic cytotoxicity (Table 1), we suggest that chlorophyll and pheophytin affect ABC-transporters, consequently reversing MDR.

In our bacterial tests series, we established an ethidium bromide-verapamil system to select bacteria for further testing based on their efflux pump activity [3]. Ethidium bromide crosses cytoplasmic membranes and subsequently intercalates DNA inside bacteria, giving rise to increased fluorescence when excited by ultraviolet light. At the same time, efflux pumps of MDR bacteria recognize this substrate and extrude it to extracellular regions. Verapamil inhibits efflux pumps and decreases ethidium bromide resistance, which could be observed by augmented ethidium bromide fluorescence (Figure 2). Based on the cartwheel method, MRSA NTCT 10,442 was selected as a representative strain for further study, as it proved sensitive to verapamil (Figure 2). This phenotype is in agreement with previous studies demonstrating that verapamil is capable of blocking NorA pumps of *S. aureus* [5]. Since chlorophyll and pheophytin contain porphyrin structures, we carried out photosensitization. When bacteria were exposed to light, the MIC of chlorophyll and its derivative decreased by at least 32-fold (Table 3). Checkerboard assays were carried out testing the antimicrobial effect of ethidium bromide when combined with chlorophyll under light and dark conditions. Our results showed that the MIC of ethidium bromide it was further reduced by a factor of 16 under light conditions, while it remained unchanged in the dark (Table 4). Recently, porphyrins have received great attention as they could be ideal candidates in photodynamic therapies by catalysing peroxidase and oxidase reactions. Besides, the absorption of photons is thought to generate reactive oxygen species (ROS) that interact with lipids of bacterial biomembranes [26]. Moreover, photodynamic antimicrobial chemotherapy (PACT) has been extensively studied; it involves three elements: oxygen, a photosensitizer, and corresponding laser light with the matching absorption wavelength of the photosensitizer [27]. Several chlorophyll related compounds were identified as photosensitizers suitable for PACT. For instance, protochlorophyllide inactivates gram-positive and gram-negative bacteria after illumination [28]. Besides, sodium chlorophyllin-induced phototoxicity and phototoxicity originating from chlorophyll derivatives of silkworm excreta has been reported [29,30].

## 4. Materials and Methods

### 4.1. Chemicals and Materials

Doxorubicin, 3-(4,5 dimethylthiazol-2-yl)-2,5-diphenyl-tetrazolium bromide (MTT, ≥98%), (±)-verapamil hydrochloride and Müller Hinton broth 2 were purchased from Sigma-Aldrich GmbH, Steinheim, Germany. Chlorophyll and pheophytin were isolated from spinach according to [31]. Hexane, acetone, RPMI1640 medium, penicillin-streptomycin, fetal bovine serum (FBS), l-glutamine, and dimethyl sulfoxide (DMSO) were purchased from Gibco^®^ Invitrogen, Darmstadt, Germany, and defibrinated sheep blood from Thermo Scientific GmbH, Schwerte, Germany. The tested species of bacteria were provided in courtesy of the Department of Infectious Diseases, Medical Microbiology and Hygiene, Heidelberg University, Heidelberg, Germany.

### 4.2. Cell Culture

Human T-cell lymphoma CCRF-CEM and its subline, CEM ADR5000, which is resistant to doxorubicin and highly expresses P-gp, were cultured in RPMI1640 medium with 10% heat-inactivated calf serum (FBS), 2 mM l-glutamine, and antibiotics (100 U/mL penicillin, 100 µg/mL streptomycin) at 37 °C, 5% CO_2_, and 95% humidity. CEM/ADR5000 cells were cultivated with 4 µg/mL doxorubicin for 24 h per week to keep a high level of P-gp expression [11,31,32].

### 4.3. Cytotoxicity Assay

For CCRF and CEM/ADR5000, 3 × 10^4^ cells/well were seeded in 96-well plates and treated with different concentrations of substances (doxorubicin, chlorophyll, and pheophytin) for 48 h. Then 0.5 mg/mL of MTT was added and incubated for 3 h. The medium was discarded, and formazan crystals were dissolved in 100 µL DMSO per well. The optical density was measured at 570 nm using a Tecan microplate reader (Crailsheim, Germany). Experiments were conducted in triplicates per plate and repeated at least three times independently [32].

Cell viability and relative resistance were determined as follows:

(1) $$\mathrm{Viability}\%=\frac{\mathrm{OD}\;\mathrm{substance}\;\mathrm{treated}\;\mathrm{cells}\;-\mathrm{OD}\;\mathrm{substance}\;\mathrm{medium}\;\mathrm{control}}{\mathrm{OD}\;\mathrm{untreated}\;\mathrm{cells}\;-\mathrm{OD}\;\mathrm{medium}\;\mathrm{control}}\%$$

(2) $$\mathrm{Relative}\;\mathrm{resistance}=\frac{{\mathrm{IC}}_{50}\;\mathrm{value}\;\mathrm{in}\;\mathrm{resistant}\;\mathrm{cell}\;\mathrm{line}\;\left(\mathrm{CEM}/\mathrm{ADR}5000\right)}{{\mathrm{IC}}_{50}\;\mathrm{value}\;\mathrm{in}\;\mathrm{sensitive}\;\mathrm{parent}\;\mathrm{cell}\;\mathrm{line}\;\left(\mathrm{CCRF}\;-\mathrm{CEM}\right)}$$

### 4.4. MDR Reversal Assay

Different concentrations of the chemotherapeutical drug doxorubicin were applied to CCRF-CEM cells and CEM/ADR5000 cells in combination with a non-toxic concentration of chlorophyll and pheophytin, which was determined prior to the tests. After 48 h incubation, MTT assay was conducted according to the cytotoxicity assay. In order to investigate the interactions between doxorubicin with chlorophyll and pheophytin, CI (combination index), RR (reversal ratio), and IB (isobologram) were calculated as follows:

(3) $$\mathrm{CI}=\frac{C_{A,X}}{{\mathrm{IC}}_{X,A}}+\frac{C_{B,X}}{{\mathrm{IC}}_{X,B}}$$

C_A,X_ and C_B,X_ are the concentrations of drug A (doxorubicin) and drug B (chlorophyll or pheophytin) used in combination to reach an IC_50_ value. ICX,A and ICX,A are the IC_50_ values for single drugs, A (doxorubicin) and B (chlorophyll or pheophytin). The combination index (CI) was used to quantitatively identify synergism (CI < 1), additive effects (CI = 1), and antagonism (CI > 1) [24,32].

The reversal ratio (RR) was defined as the IC_50_ value of doxorubicin divided by the IC50 value of doxorubicin in combination with chlorophyll or pheophytin. RR values indicate the cytotoxicity enhancement ratio, which can be used to quantify the potential power of MDR reversal agents.

The isobologram (IB) analysis evaluates the nature of the interaction of two drugs. First, the concentrations of drugs A and B required to produce a defined single-agent effect, such as IC_50_, when used as single agents, are placed on the x and y axes in a two-coordinate plot, corresponding to (C_A_, 0) and (0, C_B_), respectively. The line connecting these two points is the line of additivity. Second, the concentrations of the two drugs used in combination to provide the same effect, denoted as (C_A_, C_B_), are placed in the same plot. Synergy, additivity, or antagonism are indicated when (C_A_, C_B_) is located below, on, or above the line [24].

### 4.5. Statistical Analysis

All experiments were repeated at least three times independently. Data are presented as mean ± one standard deviation (SD). The significance of differences in the mean values was analysed using unpaired two-tailed t-test. P values < 0.05 were considered as significant. IC_50_ was defined as the concentrations required to inhibit the growth of treated cells by 50% when compared with the control, which was calculated from the dose-response curves using a four-parameter logistic fitting curve in SigmaPlot^®^ 11.0.

### 4.6. Selection of Bacteria to Investigate Effects on Efflux Pump Activity

**Figure 3:**
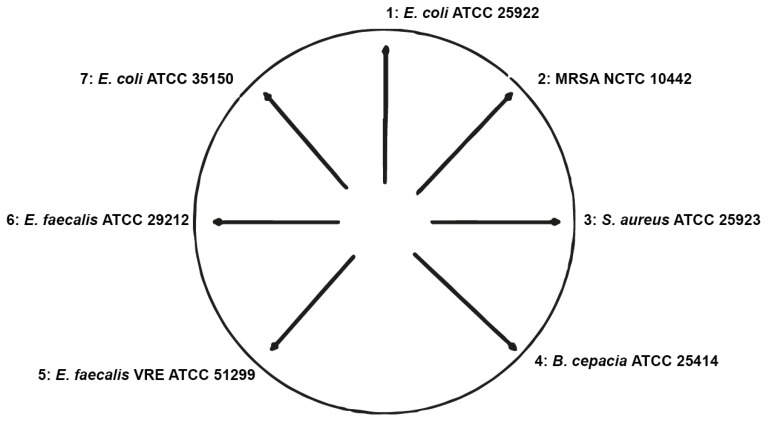
Schematic representation of the cartwheel method. Three strains of gram-negative bacteria (1, 4, 7) and four strains of gram-positive bacteria (2, 3, 5, 6) were streaked on EtBr-agar plates and incubated overnight.

Ethidium bromide agar plates were used to identify an appropriate bacterial strain for further investigation of efflux pump activities. Müller Hinton agar plates supplemented with 5% sheep blood and containing ethidium bromide at concentrations between 0 and 1.5 µg/mL, either alone or in combination with the efflux pump inhibitor verapamil (200 µg/mL), were utilized to screen *E. coli* ATCC 25922, *E. coli* ATCC 35150, *E. faecalis* ATCC 29212, *E. faecalis* VRE 51299, *B. cepacia* ATCC 25414, *Staphylococcus aureus* ATCC 25923, and methicillin-resistant *Staphylococcus aureus* (MRSA) NCTC 10442. The presence and activity of efflux pumps extruding ethidium bromide from the cells were assessed by means of the cartwheel method and included the visual inspection of the fluorescence intensity and the extent of microbial growth using a UVP GelDoc-It Imager (UVP, Upland, CA, USA) [33]. Briefly, an overnight culture of the bacteria was adjusted to an optical density of 0.5 McFarland standards. The cell suspension was streaked on ethidium bromide agar plates using sterile cotton swabs in a concentric manner (Figure 3).

### 4.7. Antimicrobial Activity: Broth Microdilution of Single Agents

**Figure 4:**
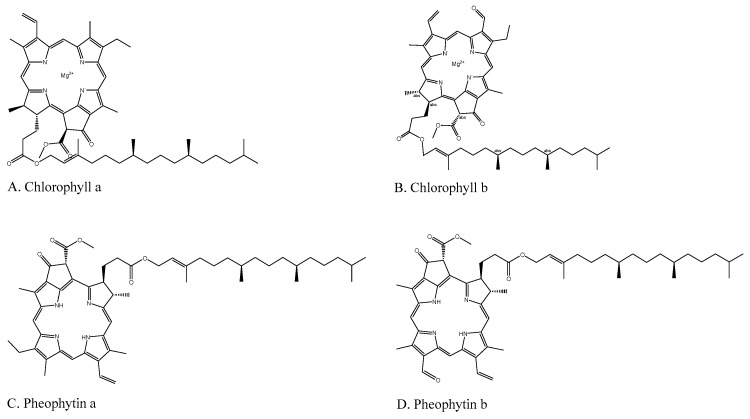
Structures of chlorophyll derivatives.

In order to identify the minimum inhibitory concentrations (MICs) for the following combination assays, broth microdilutions were performed. Ethidium bromide, verapamil, chlorophyll, and pheophytin were tested against the MRSA NTCT 10,442 that had been selected in the cartwheel method. Chlorophyll and pheophytin were stabilized in Tween-80 at concentrations ranging from 2 µg/mL to 2048 µg/mL. The maximum concentration of the stabilizer did not exceed 0.5% and did not inhibit bacterial growth as indicated by negative controls. Besides, ethidium bromide and verapamil were tested at concentrations up to 1 µg/mL and 4096 µg/mL, respectively. All organisms were cultured on Columbia Agar supplemented with 5% sheep blood and in cation-adjusted Müller-Hinton broth according to the method of CLSI (2012) with incubation at 35 °C for 18 h and 5% CO_2_ in the dark. Chlorophyll and chlorophyll-derived compounds contain photosensitive porphyrins (Figure 4), and due to the generation of reactive oxygen species [34], may have the potential to induce phototoxicity towards bacteria when exposed to visible light. For this reason, microdilutions were repeated under continuous illumination. Solvent growth controls and sample sterility controls were run in parallel on the same plates. Ampicillin served as a positive control. The tests were conducted in duplicate per plate and performed three times.

### 4.8. Synergism Between Chlorophyll and Ethidium Bromide Under Light and Dark Conditions

In checkerboard microdilution assays, synergism of the combinations of ethidium bromide and chlorophyll was tested by serial dilutions of ethidium bromide (0.031–2 µg/mL) and chlorophyll (2–2048 µg/mL) using MRSA NTCT 10,442, which had been shown to be a suitable candidate in the cartwheel and broth microdilution tests (see above). Conditions of incubation were the same as in the microdilution assays. The tests were carried out with and without illumination, as mentioned before.

## 5. Conclusion

In conclusion, chlorophyll and pheophytin from spinach are able to inhibit or reverse multi-drug resistance in cancer cells and bacteria. This effect is probably mediated by the inhibition of ABC-transporters and might open a new avenue for the treatment of cancer and infectious diseases. This proposal needs to be evaluated in in vivo studies.

## Figures and Tables

**Table 1:** The cytotoxicity IC_50_ and relative resistance of CCRF-CEM cells and multi-drug resistant CEM/ADR cells against chlorophyll, pheophytin, and doxorubicin (positive control). Data represent means ± SD.

Drug	IC_50_ in CCRF-CEM	IC_50_ in CEM/ADR5000	Relative Resistance
**Doxorubicin** (µM)	0.34 ± 0.032	95.76 ± 8.495	281.65
**Pheophytin** (µg/mL)	69.50 ± 3.617	83.42 ± 10.516	1.2
**Chlorophyll** (µg/mL)	167.44 ± 15.696	>256	>1.5

**Table 2:** The cytotoxicity of doxorubicin against CEM/ADR cells and CCRF-CEM, either alone or in combination with chlorophyll or pheophytin. Data were obtained from at least three independent experiments and are represented as mean ± SD. Combination index (CI) 0.1–0.3: strong synergism and 0.3–0.7: synergism. RR: reversal ratio, CI: combination index.

	CEM/ADR5000 Cell				CCRF-CEM Cell			
Doxorubicin	IC_50_ (µM of Dox)	RR	CI	Interpretation	IC_50_ (µM of Dox)	RR	CI	Interpretation
alone	95.76 ± 8.495	1	NR	not relevant	0.34 ± 0.032	1	NR	not relevant
+10 µg/mL Pheophytin	30.57 ± 4.984	3.13	0.438	synergism	0.34 ± 0.027	1	NR	not relevant
+10 µg/mL Chlorophyll	35.19 ± 4.789	2.72	<0.407	synergism	0.35 ± 0.050	1	NR	not relevant

**Table 3:** Comparison of the minimum inhibitory concentrations (MICs) of chlorophyll and pheophytin when incubated with MRSA NCTC 10,442 in a dark and light environment.

Drug	MIC (µg/mL)	
	Dark	Light
**Chlorophyll**	>2048	64
**Pheophytin**	>2048	128
**Verapamil**	512	512
**EtBr**	>1	>1
**Ampicillin**	8	8

**Table 4:** Results of the combinations assays using ethidium bromide and chlorophyll against MRSA NCTC 10442. All values are given in µg/mL.

Cond.	MIC Chloro	MIC EtBr	MIC Chloro + EtBr	MIC EtBr + Chloro	FIC Chloro	FIC EtBr	FICI	Int.
**Light**	64	2	4	0.03	0.06	0.02	0.08	SYN
**Dark**	>2048	2	>2048	2	1	1	2	IND

Cond.: condition, Chloro: chlorophyll, EtBr: ethidium bromide, FIC: fractional inhibitory concentration, FICI: fractional inhibitory concentration index, Int.: Interpretation based on the FICI (synergism: FICI ≤ 0.5, indifference: 0.5 < FICI ≤ 4). MIC Chloro + EtBr: MIC of chlorophyll when combined with ethidium bromide. MIC EtBr + Chloro: MIC of ethidium bromide when combined with chlorophyll.
